# Optimizing Genomic Methods for Mapping and Identification of Candidate Variants in ENU Mutagenesis Screens Using Inbred Mice

**DOI:** 10.1534/g3.117.300292

**Published:** 2017-12-05

**Authors:** Krista A. Geister, Andrew E. Timms, David R. Beier

**Affiliations:** * Center for Developmental Biology and Regenerative Medicine, Seattle Children’s Research Institute, Seattle, Washington 98101; † Department of Pediatrics, University of Washington School of Medicine, Seattle, Washington 98195

**Keywords:** *Mus musculus*, *Colgalt1*, *Plod3*, *Kif20b*, *Tgds*, *Fbn2*, Mutant screen report

## Abstract

Positional cloning of ENU-induced mutations has traditionally relied on analysis of polymorphic variation between two strains. In contrast, the application of whole-genome sequencing (WGS) has enabled gene discovery in mutant lines maintained on an inbred genetic background. This approach utilizes genetic variation derived from ENU-induced variants for mapping and reduces the likelihood of phenotypic variation, making it an ideal method for genetic modifier screening. Here, we describe the results of such a screen, wherein we determined the minimal number of mutant genomic DNA samples to include in our analyses and improved the sensitivity of our screen by individually barcoding each genomic DNA library. We present several unique cases to illustrate this approach’s efficacy, including the discovery of two distinct mutations that generate essentially identical mutant phenotypes, the ascertainment of a non-ENU-induced candidate variant through homozygosity mapping, and an approach for the identification of putative dominant genetic modifiers.

Historically, mutagenesis screens in the mouse have relied on polymorphic variation between strains to facilitate homozygosity mapping. This strategy requires an outcross, which may involve a great deal of time, breeding, and effort. After mapping the locus, one may need to rely on beneficial recombination in subsequent affected individuals to narrow the interval, while searching for ENU-induced mutations by individually sequencing exons within the locus. This method also does not lend itself particularly well to genetic modifier screening, as the polymorphic variation from the outcross strain may add additional complexity to both the phenotype and the genetic analysis. Our laboratory (Gallego-Llamas *et al.* 2015) and others (Bull *et al.* 2013; Simon *et al.* 2015; Caruana *et al.* 2013) have developed strategies that use next-generation sequencing technologies to accelerate homozygosity mapping, variant calling, and confirmation on an inbred background. Doing so minimizes phenotypic variation and limits genetic variation to ENU-induced variants. We sought to optimize this approach for the discovery of genetic modifiers of developmental phenotypes.

The traditional outcross has been similarly optimized to provide fine mapping of the locus with a minimal number of markers (Moran *et al.* 2006). ENU induces roughly 3000 heritable variants per genome (Quwailid *et al.* 2004); ascertainment of even half that number of variants is more than enough to map a causal locus (Moran *et al.* 2006). Our bioinformatics analysis of mutagenized inbred lines involves dividing the genome into windows of a specified size and calculating the following for each window: the number of homozygous SNPs, percentage of homozygous SNPs, and the percentage of nonreference alleles, which we call the novel allele frequency (Gallego-Llamas *et al.* 2015). Loci with a high number and percentage of homozygous SNPs and a high novel allele frequency represent regions that contain a concentration of homozygous ENU-induced variants. It is worth noting that the variants used to map the locus will likely include the causal mutation. We focused our analysis on variants that occur in coding regions or splice sites and conducted Sanger sequencing on additional affected embryonic DNA samples to identify the causal variant.

Our initial attempt to map and ascertain ENU-induced variants using this method was successful and yielded valuable insights (Gallego-Llamas *et al.* 2015). First, we found that WGS provided better mapping resolution than whole-exome sequencing. Second, pooling samples without individually barcoding them can complicate the analysis. We conducted WGS on individually barcoded sample libraries in all the work described in this report. This led to some additional insights regarding the sensitivity, cost-effectiveness, and power of our approach. We discovered five mutant alleles: two of which have not been previously described, and three that are remutations of previously characterized mutant loci (Rautavuoma *et al.* 2004; Ruotsalainen *et al.* 2006; Dwyer *et al.* 2011; Johnson *et al.* 1998; Arteaga-Solis *et al.* 2001; Browning *et al.* 2001; Chaudhry *et al.* 2001; Miller *et al.* 2010; Shi *et al.* 2013). We have successfully mapped loci using as few as two mutant samples, we have learned that this technique is sensitive enough to identify two independent mutations that cause what was thought to be one mutant phenotype, and we have used this method to clone a non-ENU-induced mutant locus. Additionally, this strategy can be used to query putative modifying loci regardless of inheritance pattern.

## Materials and Methods

### Generation of mutant mice

All animal work was reviewed and approved by the Seattle Children’s Research Institute’s (SCRI) Institutional Care and Use Committee. ENU mutagenesis was carried out by George Carlson’s laboratory at the McLaughlin Research Institute (Great Falls, MT) as previously described (Gallego-Llamas *et al.* 2015; Stottmann and Beier 2014). G1 male mice were shipped to Seattle Children’s Research Institute and bred to C57BL/6J females. G2 daughters were backcrossed to the G1 male and were checked daily for the presence of a copulation plug. Embryos were screened at either E17.5 or E18.5 for mutant phenotypes.

### Genomic DNA isolation

Genomic DNA for WGS was extracted from mutant embryonic liver samples using the QIAGEN DNeasy Blood and Tissue Kit (#69504) according to the manufacturer’s instructions, but with some modifications. Approximately 10 mg of embryonic liver was used, and was snipped into very small pieces in individual plastic weigh boats with fine dissecting scissors. Lysis buffer was used to collect all of the tissue from the weigh boat. Samples were digested with the Proteinase K provided in the kit (20 mg/ml) for 1 hr, with vortexing every 10 min. RNA was digested with 8 μl of RNase A (QIAGEN #19101, 100 mg/ml at 7000 units/ml) for 2 min. Two elutions were conducted into two separate microfuge tubes, with elution buffer incubated on the column for 5 min before centrifugation. DNA was tested at SCRI for purity and quality using a Nanodrop spectrophotometer and the Qubit dsDNA Broad Range Assay (#Q32850; Thermo Fisher Scientific). DNA samples were shipped to HudsonAlpha Genomic Services Laboratory (Huntsville, AL) or NovoGene (Chula Vista, CA), for WGS.

### Homozyosity mapping

Mapping, variant calling, and homozygosity mapping were performed as in our previous publication (Gallego-Llamas *et al.* 2015). In summary, we divided the genome in windows and identified high-quality ENU SNPs. We subsequently counted the number of homozygous variants, percentage of homozygous variants, and average nonreference allele frequencies for each window. To identify potential casual variants, we identified all coding and splice site (within 10 bp of an exon) homozygous mutations of good quality.

### Heterozygous modifier identification

We used two approaches to identify ENU-induced dominant modifiers, either using individual mice or a combination of all reads for a phenotype. For individual mice, we identified ENU SNPs by eliminating from further consideration those in dbSNP, Sanger mouse strains, in-house pedigrees, or repeat regions. We then identified SNPs that had total coverage of five reads or greater per individual sample, and had either a heterozygous or homozygous mutant genotype in all mice analyzed (five individuals for cleft palate and three individuals for exencephaly). For the combined sets, we identified high-quality ENU SNPs by; removing SNPS if they were in dbSNP, a Sanger mouse strain, an in-house unrelated pedigree, or a repeat region. We also required SNPs to have a quality score of ≥ 30 and coverage of five or more reads. We then removed SNPs if in other “*fosse*” types, *i.e.*, exencephaly or cleft palate, and then counted the number of SNPs per sliding window (10 Mb with a 1 Mb overlap).

### Validation of candidate variants

Primers were designed to amplify regions containing our variants of interest using Primer-BLAST (NCBI, Bethesda, MD). We conducted Sanger sequencing on these PCR products amplified from additional affected embryonic DNA samples (Eurofins Genomics, Louisville, KY). Sanger sequencing data were analyzed using FinchTV (Geospiza, Seattle, WA), 4Peaks (Nucleobytes, Aalsmer, The Netherlands), Sequencher 5.0 (Gene Codes Corp., Ann Arbor, MI), and/or the Lasergene 12 Suite (DNAStar, Madison, WI).

### Skeletal preparations

Skeletal preparations were performed as previously described (Ovchinnikov 2009).

### Data availability

Strains are available upon request. Sequence data are available in the BioProject database via BioProject ID: PRJNA412014. Supplemental Material, File S1 contains code used for homozygosity mapping.

## Results and Discussion

### Mutant phenotypes obtained and sequencing analysis

**Figure 1:**
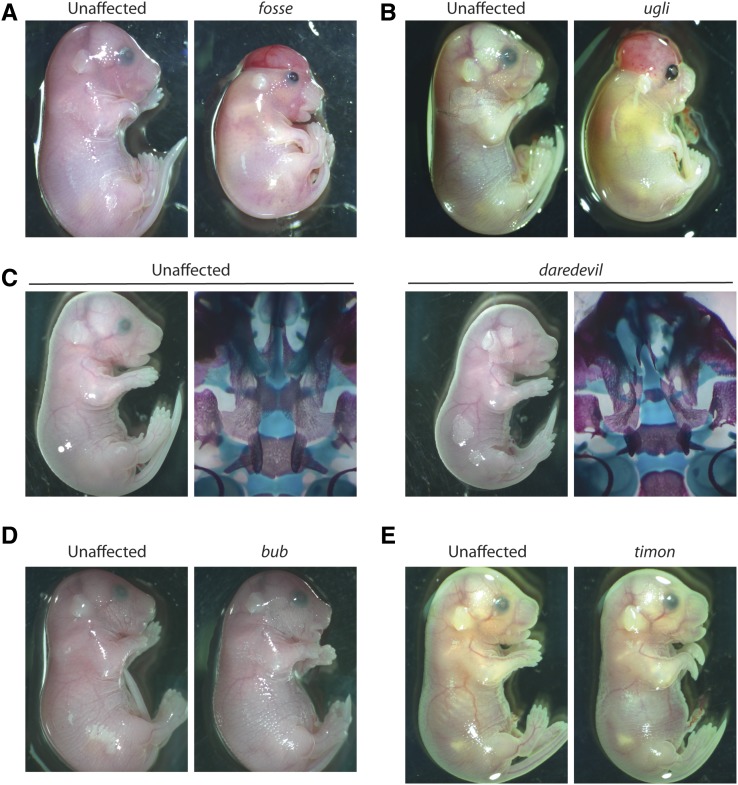
Mutant phenotypes discovered by ENU mutagenesis. (A) The *fosse* phenotype exhibits bent wrists, eye defects, skeletal defects, and variable incidence of cleft palate and exencephaly. (B) The *ugli* phenotype closely resembles the *fosse* phenotype, even with regard to cleft palate and exencephaly. (C) The *daredevil* phenotype is characterized by bilateral anophthalmia, growth insufficiency, and cleft palate. (D) *bub* mutant embryos display severe micrognathia and cleft palate. (E) Bent wrists, micrognathia, eye and ear defects, and hindlimb syndactyly are hallmarks of the *timon* mutant phenotype. ENU, N-ethyl-N-nitrosourea.

We selected the C57BL/6J background for our inbred screen and carried out a standard G2 backcross breeding scheme as previously described (Gallego-Llamas *et al.* 2015). In addition, the founders of these mutant lines were offspring of our previously described sequential dosage pipeline (Gallego-Llamas *et al.* 2016), which resulted in many of our founder males belonging to one of several pedigrees. Mutant lines where multiple affected embryos were observed in more than one litter were selected for further study. Five mutant phenotypes were discovered (Figure 1). Together, they represent a collection with craniofacial, eye, and digital anomalies. Embryonic liver served as the source of genomic DNA for WGS, while liver and tail were used for other downstream applications, including PCR amplification followed by Sanger sequencing. Once more than eight embryos with these phenotypes had been collected, their genomic DNA was extracted and the samples with the highest yield and best A_260_:A_280_ ratio were selected for WGS, followed by homozygosity mapping to map ENU-induced homozygous loci and variant calling to determine likely candidate variants.

### Homozygosity mapping can distinguish two mutations that cause nearly identical phenotypes

Two of the mutant phenotypes (Figure 1, A and B, *fosse* and *ugli*), were impossible to distinguish from one another prior to sequencing analysis. Three founder males from the same ENU pedigree produced embryonic progeny with these phenotypes. Due to their shared lineage, we initially presumed that this phenotype was caused by a single mutation. Embryos exhibited bent wrists, open eyelids, eye defects, edema, short rib cages, and growth insufficiency. We named this presumed single mutant phenotype *fosse*. A total of eight genomic DNA samples from *fosse* embryos from two of the three founder males were used for library preparation and WGS analysis. All samples were individually barcoded to allow us to separate each individual library’s results from the others, if necessary.

**Figure 2:**
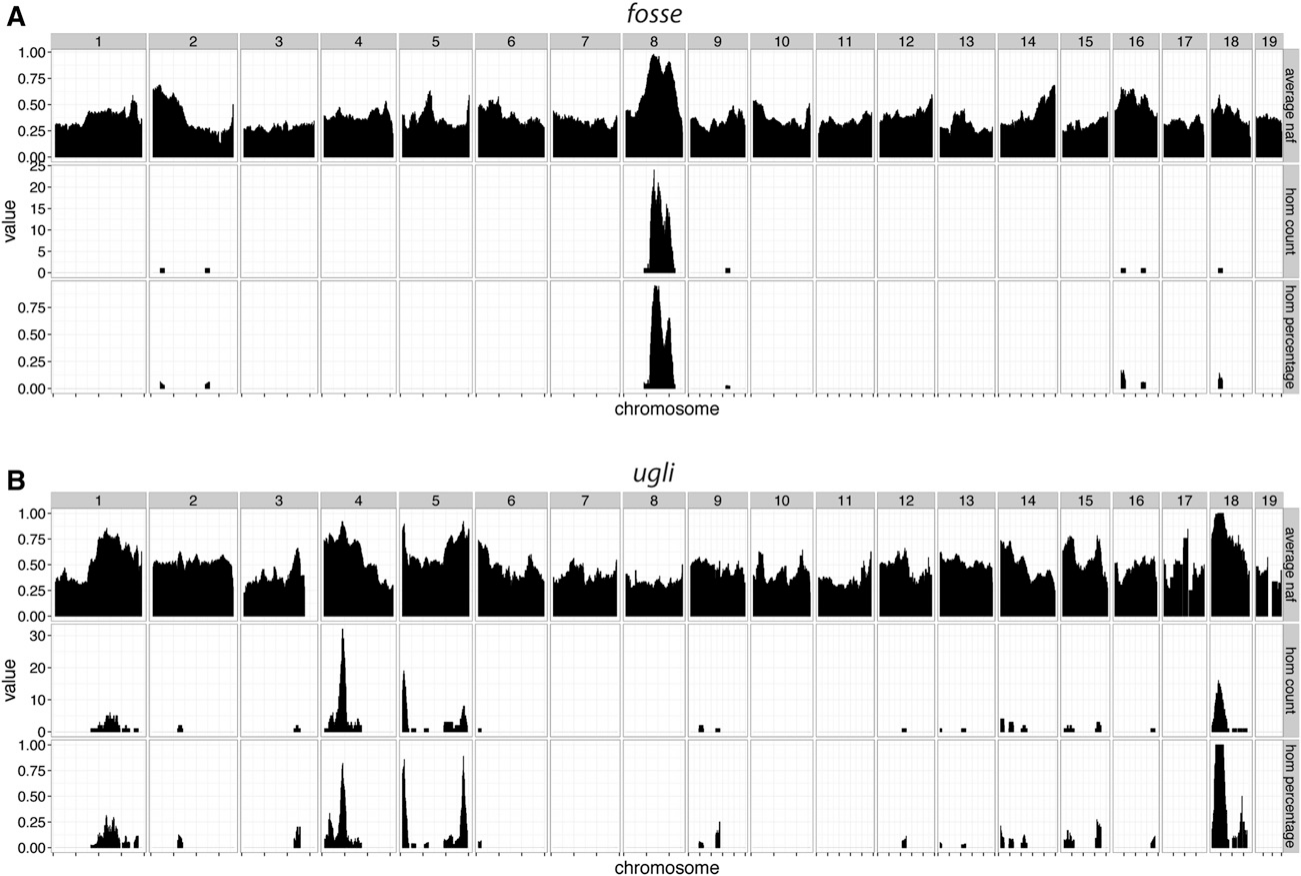
Homozygosity mapping of *fosse* and *ugli* reveals distinct causal genetic loci associated with these two highly similar phenotypes. (A) Our homozygosity mapping of six affected individuals indicates that the *fosse* causal variant is located on chromosome 8. (B) Our remaining two samples’ combined homozygosity mapping results highlighted homozygous regions of interest on chromosomes 4, 5, and 18. Both maps were generated using windows 10 Mb in size with a 1 Mb overlap to calculate all three variables shown. Average naf indicates the average frequency of the nonreference allele in all putative ENU SNPs within a window. Hom count refers to the number of homozygous SNPs within each window, whereas hom percentage is the percentage of SNPs that were called homozygous. Regions of interest should have high average naf (likely ENU-induced), and high homozygous counts and percentages. ENU, N-ethyl-N-nitrosourea; hom, homozygous; naf, novel allele frequency; SNP, single nucleotide polymorphism.

We were unable to map the *fosse* locus when all eight samples were included in our sequencing analysis. Multiple iterations of our analysis with different combinations of samples were conducted, which ultimately revealed that the presumptive *fosse* phenotype was, in fact, two nearly identical mutant phenotypes (Figure 1, A and B) caused by two independent mutations (Tables S1 and S2 in File S1). Six of our *fosse* samples had a common homozygous region on mouse chromosome 8 (Figure 2A), and the two remaining samples shared several regions of homozygosity on mouse chromosomes 4, 5, and 18 (Figure 2B). We chose to name the phenotype of these two outliers *ugli*.

After our mapping analysis, we restricted our focus to variants in all homozygous regions identified that were predicted to occur in coding regions and near splice sites. This generated the list of variants in Tables S1 and S2 in File S1. While it is possible that a causal mutation will occur in nontranscribed sequences, there is presently only one report documenting an ENU-induced mutation occurring in a regulatory region (Sagai *et al.* 2004) and a single report describing an ENU-induced mutation in a microRNA seed region (Lewis *et al.* 2009). There may be bias in the conclusion that exons and their flanking regions are likely to contain the mutated nucleotide, as nontranscribed regions are often not examined in detail. However, in the characterization of ENU mutants mapped to loci where known candidates reside, causal sequence variants can usually be found in coding regions or splice sites [*e.g.*, Hart *et al.* (2005)].

Analysis of the homozygous regions in the two *ugli* mutants identified a missense mutation in *Plod3* as the only coding/splice site variant. Additional genotyping confirmed that the variant in *Plod3* was homozygous in all affected embryos (Table S2 in File S1). Several variants on chromosome 8 were analyzed to determine the *fosse* candidate mutation (Table S1 in File S1). All mutant embryos were homozygous for variants in *Colgalt1* and *Jak3*, which are 65 kb apart. However, the *Jak3* null phenotype is compatible with postnatal life (Park *et al.* 1995) and the *fosse* mutant phenotype is embryonic lethal.

This leads us to conclude that the *fosse* phenotype is caused by the missense variant in *Colgalt1*, and that the *ugli* phenotype is caused by the missense variant in *Plod3*; this is supported by the functional relatedness of these two genes. *Colgalt1* and *Plod3* both encode enzymes involved in the post-translational modification of collagens [reviewed in Yamauchi and Sricholpech (2012)]. *Colgalt1* is a galactosyltransferase and has been shown to act *in vitro* on hydroxylysines in collagen types I–V (Schegg *et al.* 2009). A *Colgalt1* null mouse phenotype has not been described, but there are *Plod3* mutant mouse lines with an array of phenotypes (Rautavuoma *et al.* 2004; Ruotsalainen *et al.* 2006). *Plod3* can act as a lysyl hydroxylase and a galactosylhydroxylysine glucosyltransferase (GGT) (Heikkinen *et al.* 2000; Wang *et al.* 2002a,b). The GGT activity of *Plod3* is critical for collagen type IV deposition and function *in vivo* (Rautavuoma *et al.* 2004; Ruotsalainen *et al.* 2006). Most importantly, *Plod3* mutations that result in either complete loss of function or loss of GGT activity result in embryonic lethality (Rautavuoma *et al.* 2004; Ruotsalainen *et al.* 2006). Our *Plod3^ugli/ugli^* embryos die in either late gestation or neonatally. Mutations in *PLOD3* have been documented in humans with lysyl hydroxylase 3 deficiency (bone fragility with contractures, arterial rupture, and deafness) (Salo *et al.* 2008). The similarities in phenotype between our *Plod3^ugli/ugli^* mutant and previously described *Plod3* mutant phenotypes (Rautavuoma *et al.* 2004; Ruotsalainen *et al.* 2006), as well as the nearly identical phenotypes observed in *Colgalt1^fosse/fosse^* and *Plod3^ugli/ugli^* embryos, provide support that we have identified the candidate mutations in both cases.

### Identification of two additional candidate ENU-induced variants from WGS of three individually barcoded samples

Given the time and numbers of mice required to discover mutant strains, as well as the cost of WGS, any strategies that can accelerate and ensure the identification of the causal variant are invaluable. We included a total of six *Colgalt1^fosse/fosse^* samples and two *Plod3^ugli/ugli^* samples in our WGS analysis, and successfully identified the candidate variant in each case. This led us to test *in silico* and in actuality how many samples are required to ensure successful identification of a causative variant. Using our data from the *Colgalt1^fosse/fosse^* WGS analysis, we randomly combined different samples and different numbers of samples, and were able to generate homozygosity maps that matched our *Colgalt1^fosse/fosse^* mapping data with only three samples (data not shown). Importantly, this number also ensured that we had enough coverage from each individual’s library to call a true variant.

**Figure 3:**
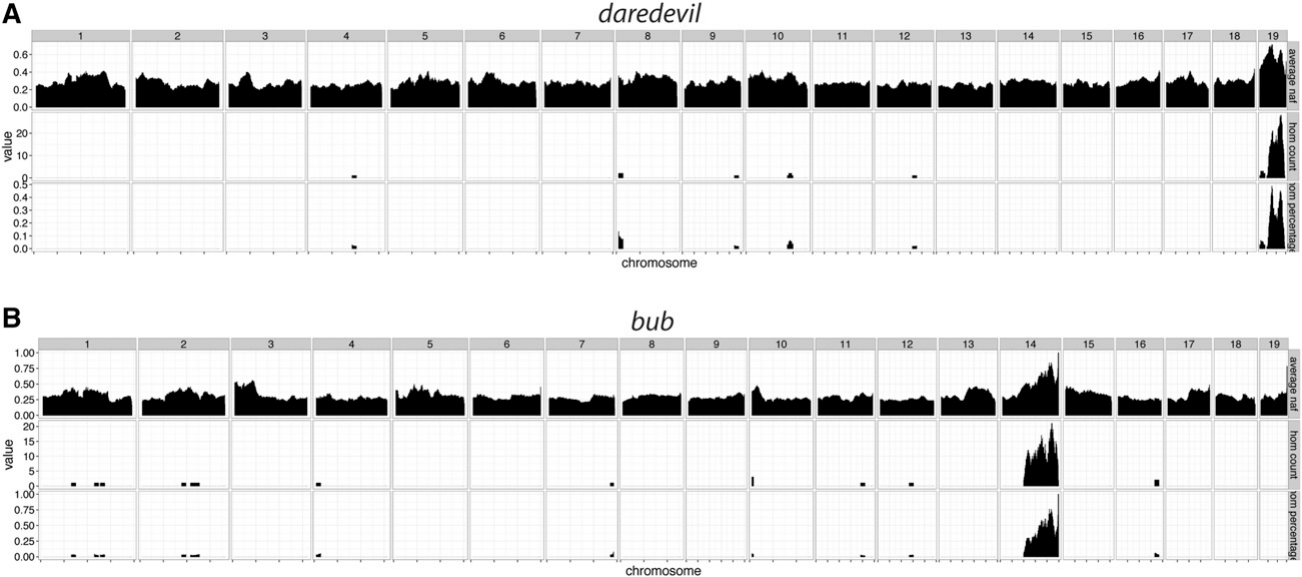
Homozygosity mapping of *daredevil* and *bub* uncovers causal loci on chromosomes 19 and 14. (A) A single homozygous nonreference peak exists for *daredevil* on chromosome 19. (B) Likewise, the *bub* homozygosity map indicates that the causal locus is on chromosome 14. Both maps were generated using 10 Mb windows with a 1 Mb overlap.

To test this in practice, we included three samples from two mutant lines that we ascertained, which we named *daredevil* and *bub* (Figure 1, C and D). Both mutants exhibit cleft palate and a number of other phenotypic abnormalities. Our homozygosity mapping from both of these mutants determined that *daredevil* mapped to chromosome 19 and *bub* mapped to chromosome 14 (Figure 3, A and B). There were only small numbers of coding region/splice sites variants in these regions (Table S3 in File S1), and we confirmed the genetic causes for both mutants by genotyping additional affected embryos (Table S3 in File S1). The *bub* phenotype is due to a variant in *Tgds*, and *daredevil* is caused by a mutation in *Kif20b*. The *Kif20b^daredevil/daredevil^* mutant phenocopies the *Kif20b^magoo/magoo^* mutant (Dwyer *et al.* 2011). *TGDS* mutations have been identified in human patients with Catel–Mantzke syndrome, which causes cleft palate and micrognathia (Ehmke *et al.* 2014), phenotypic hallmarks of the bub *Tgds^bub/bub^* mutant phenotype. These observations support our conclusion that both of these variants are likely candidates for each of these mutant phenotypes, and illustrate that we are able to reliably identify these variants using only three genomic DNA samples.

### Identification of a non-ENU-induced candidate mutation through homozygosity mapping and individual sequencing read analysis

**Figure 4:**
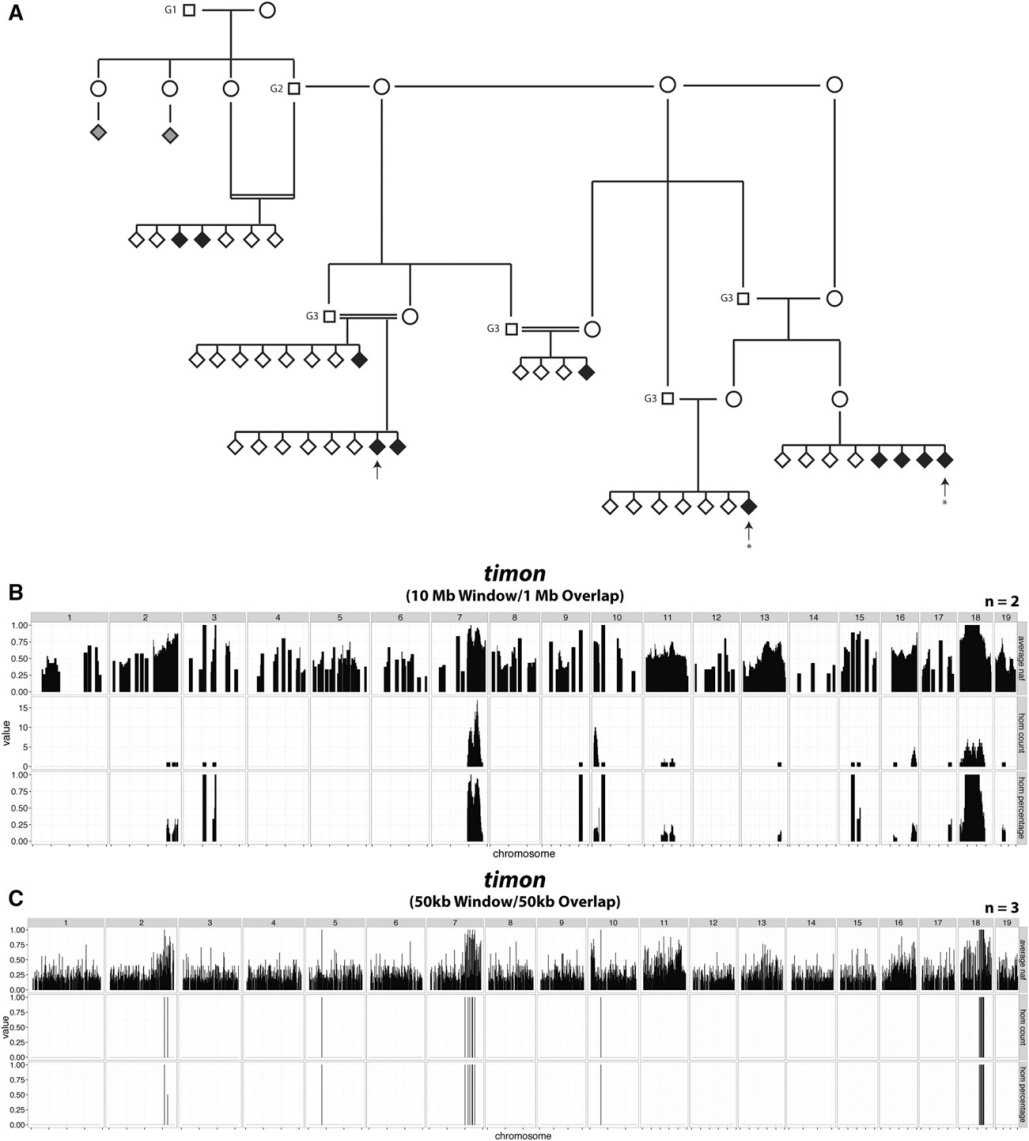
Pedigree and homozygosity mapping of *timon* indicates autosomal recessive inheritance and a causal locus on mouse chromosome 18. (A) The *timon* pedigree indicates two distinct branches: one that is more closely related to the G1 and one that is farther removed. Two individuals (gray diamonds) exhibited more severe craniofacial and limb phenotypes, and were excluded as affected by genotyping. Therefore, these two embryos represent a mutant phenotype distinct from *timon*. Arrows, genomic DNA samples selected for WGS. Asterisks: two individuals used to map the critical interval in (B). (B) The *timon* causal locus was mapped to chromosome 18 using WGS data from two of the three samples submitted. A peak on chromosome 7 was eliminated by genotyping affected individuals across the critical interval for candidate ENU-induced variants (Table S4 in File S1). (C) Reducing the window and overlap size to 50 kb in all three individuals’ mapping analysis revealed that the third sample also possessed the homozygous peak on chromosome 18. ENU, N-ethyl-N-nitrosourea; WGS, whole-genome sequencing.

*Tdgs*^bub^ and *Kif20b^daredevil^* illustrate our ability to map and ascertain causal mutations from only three samples. Our analysis of one of our mutants (*timon*, Figure 1E), led to the identification of a non-ENU-induced candidate variant. The pedigree of this line was rather complex, given that the G1 male and his only G2 male offspring had reduced fertility (Figure 4A). To secure the line, progeny testing from brother–sister matings was successfully utilized to identify obligate carriers, and the carrier males obtained were fertile. Three affected individuals’ genomic DNA samples were sent for WGS (Figure 4A, arrows). However, the homozygosity map that we generated from all three individuals’ data did not yield a clear region of interest. Analysis of a map generated from two of the more closely related individuals’ data (Figure 4A, asterisks) identified two homozygous regions, one on chromosome 7 and one on chromosome 18 (Figure 4B). Genotyping of additional affected embryos across these two intervals allowed us to exclude the locus on chromosome 7, and indicated that the causal mutation was in the distal portion of the interval on chromosome 18 (Table S4 in File S1). Importantly, examination of the third sequenced mutant confirmed that it also carried homozygous variants on distal chromosome 18. However, a recombination event occurred in the region that resulted in an interval in all three affected mice that was smaller than the window used for homozygosity mapping. While this initially confounded our mapping analysis, it ultimately facilitated the localization of the likely candidate mutation. In fact, reduction of window size uncovered the homozygous region; however, this was at the expense of marker numbers (Figure 4C).

Of the ENU variants in the recombinant interval, both coding and noncoding, none appeared to explain the mutant phenotype. We tested the effect of an intronic variant in *Pdgfrb* on the mRNA transcript via RT-PCR followed by Sanger sequencing, but the transcript sequence was normal (data not shown). This suggested the possibility that the causal variant was not induced by ENU, but was a spontaneous mutation of some kind.

**Figure 5:**
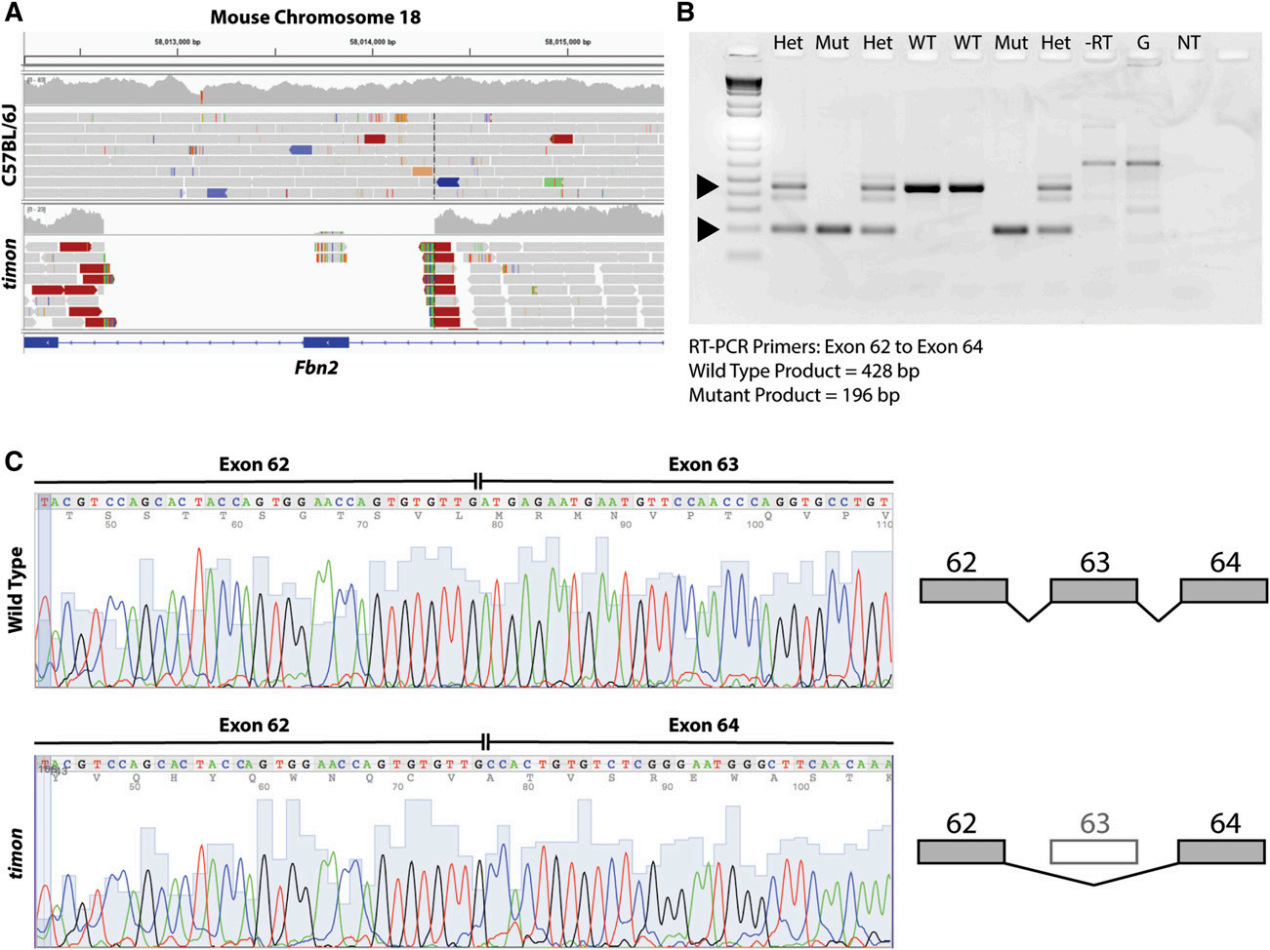
The *timon* mutant phenotype is due to deletion of exon 63 in *Fbn2*. (A) After variant calling and genotyping for individual ENU-induced variants within the interval, analysis of split reads uncovered a 1696 bp deletion that encompasses exon 63 of *Fbn2*. (B) RT-PCR of *Fbn2* transcripts using primers from exon 62–64 reveals a Mut product of 196 bp instead of 428 bp, as seen in WT and Het samples. The band in the RT reaction coincides with a band of similar size in the genomic DNA reaction (G), so this band likely reflects the presence of trace amounts of genomic DNA in our RT reactions. (C) Sequencing of RT-PCR products from WT and homozygous mutant samples demonstrate the complete loss of exon 63 sequence from the transcript. This results in frameshift and stop codons in exon 64. ENU, N-ethyl-N-nitrosourea; Het, heterozygous; Mut, mutant; NT, no template control; RT-PCR, reverse transcriptase polymerase chain reaction; WT, wild-type.

To identify a larger insertion or deletion, we identified all reads within the recombination region that had been trimmed, *i.e.*, the full read did not map at the same location. We examined all positions where we saw more than five reads with the same trimmed location. After comparing with C57BL/6J in the Integrative Genomics Viewer (Robinson *et al.* 2011), we identified a region in all three *timon* samples with split reads (Figure 5A and Table S4 in File S1), indicating that the region is deleted in the mutant genome. The *timon* deletion (chromosome 18: 58,012,626–58,014,322) is ∼1.7 kb in length, and includes exon 63 of the 65 total exons in the gene *Fbn2*, which encodes fibrillin 2. RT-PCR followed by gel electrophoresis (Figure 5B) and Sanger sequencing (Figure 5C) of mutant cDNA from exons 62–64 of *Fbn2* revealed improper splicing of exon 62 into exon 64. The loss of exon 63 in the mutant transcript results in a premature stop codon at the end of exon 64. Other mutant phenotypes due to mutations in *Fbn2* have been described, and many have the same anomalies as our *timon* mutant: hindlimb syndactyly, flexure of the wrists, and eye malformations (Johnson *et al.* 1998; Arteaga-Solis *et al.* 2001; Browning *et al.* 2001; Chaudhry *et al.* 2001; Miller *et al.* 2010; Shi *et al.* 2013).

This is an interesting case where ENU-induced variants around the deletion served as genetic markers that allowed us to map the candidate locus and mutation. For future analyses, it is worth keeping in mind that mutations ascertained in an ENU mutagenesis screen may not be ENU-induced. By conducting WGS, one is able to readily probe the entire genome for these types of mutations, should the need arise, as it did with *Fbn2^timon^*.

### Inbred screening strategy can be used to query for dominant-acting modifying loci

In addition to the phenotypic features listed above, 18% of *fosse* embryos presented with cleft palate and 26% presented with exencephaly. These phenotypes were never found in unaffected siblings. A χ^2^ analysis indicated that this result was highly significant, demonstrating that these features were contingent on homozygosity for the *fosse* allele and were not due to independently acting mutations (Table 1). As previously stated, our inbred screening strategy was designed with the intent that it could be used to identify genetic modifiers, so we attempted to assess whether the variable expressivity of cleft palate and exencephaly in *Colgalt1^fosse/fosse^* mutants was due to genetic interactions.

**Table 1:** χ^2^ analysis of cleft palate and exencephaly indicates that both phenotypes are contingent with the *fosse* mutant phenotype

Phenotype	*fosse* Observed	Wild-Type Expected	Wild-Type Observed
Unaffected	51 (82.3%)	128	155
Cleft Palate	11 (17.7%)	27	0*^a^*
			*^a^**P* < 0.0001
Unaffected	46 (74.2%)	115	155
Exencephaly	16 (25.8%)	40	0*^a^*
			*^a^**P* = 0.0009
Total Embryos	62		155

a *P*-values indicate that the lack of cleft palate and exencephaly in unaffected littermates is a significant deviation from ratio observed among the *fosse* cohort (percentages in parentheses).

Our initial WGS analysis of *fosse* mutants included five embryos with cleft palate and one with exencephaly. For the modifier analysis, we added two additional *fosse* embryos with exencephaly. For both cases, we identified only a single region of homozygosity (corresponding to the *Colgalt1* locus), excluding the possibility that these phenotypes were due to unlinked recessive-acting modifying mutations.

**Figure 6:**
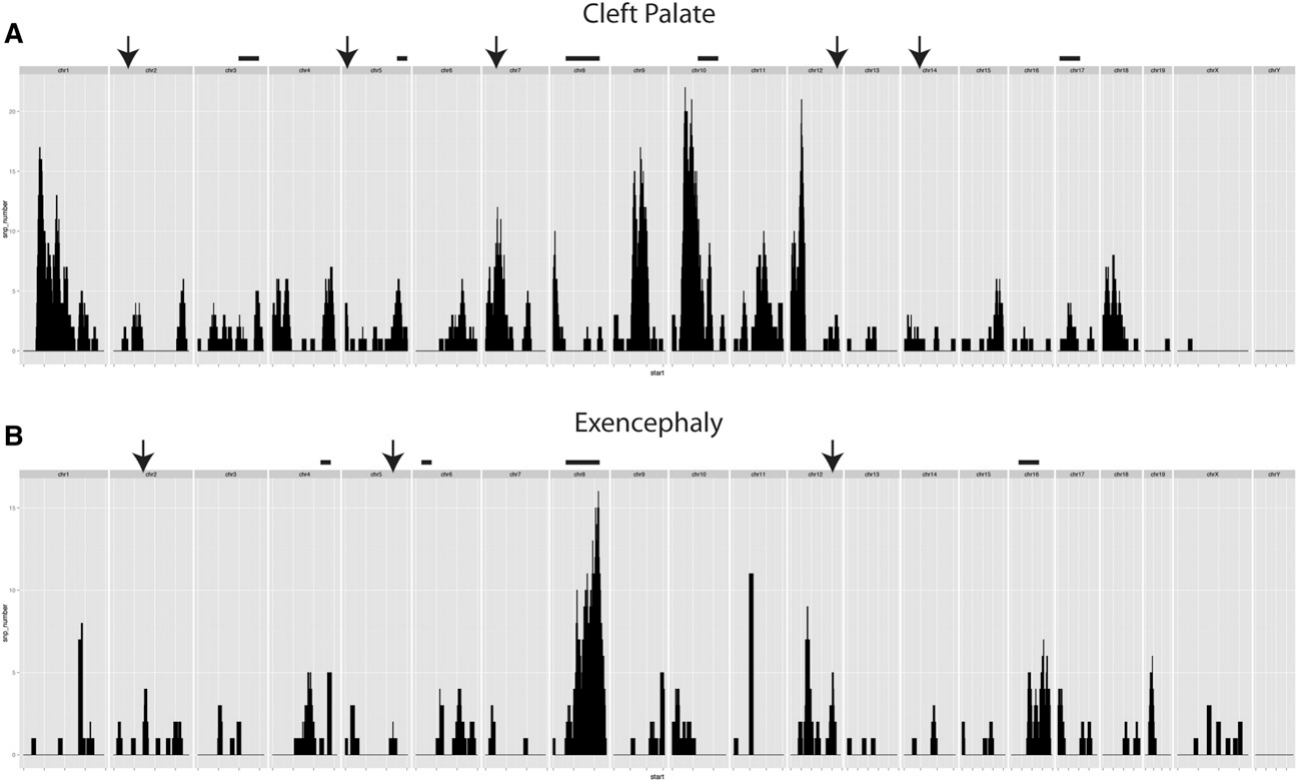
Filtering of variants by phenotype generates a map of possible modifying loci contributing to cleft palate or exencephaly. (A) Number of SNPs in each 10 Mb window (1 Mb overlap) specific to samples with cleft palate (*n* = 5). Arrows indicate regions where one SNP was tested for association, bars indicate a locus where more than one SNP has been tested. (B) The same parameters used in (A) were applied to the sequencing data from our samples with exencephaly (*n* = 3). SNP, single nucleotide polymorphism.

Therefore, we decided to test whether cleft palate may be due to a dominant-acting modifier that, when combined with homozygosity for the *Colgalt1*^fosse^ variant, would generate an embryo with cleft palate or exencephaly. We first focused our analysis on heterozygous ENU-induced variants that were shared between the five cleft palate samples, but were not present in the heterozygous state in our three samples with exencephaly. This resulted in the identification of 22 variants to consider for validation. After genotyping an additional four samples with cleft palate for all of these variants, as well as multiple fosse embryos without cleft palate, we concluded that cleft palate does not appear to be due to a unique heterozygous variant in our *Colgalt1^fosse/fosse^* embryos (Figure 6A and data not shown).

For the exencephaly phenotype, we identified 10 dominant modifier candidates, and genotyped an additional four individuals with and without exencephaly to determine if any of them were causal. None of these variants were associated with exencephaly (Figure 6B and data not shown). These data demonstrate that the exencephaly phenotype in *Colgalt1^fosse/fosse^* embryos, like cleft palate, is probably due to stochastic processes during embryonic development and not to heritable genetic interactions.

In conclusion, we have successfully identified the causal variants for five mutant lines, three of which are remutations of previously characterized loci [*Plod3^ugli/ugli^* (Rautavuoma *et al.* 2004; Ruotsalainen *et al.* 2006), *Kif20b^daredevil/daredevil^* (Dwyer *et al.* 2011), and *Fbn2^timon/timon^* (Johnson *et al.* 1998; Arteaga-Solis *et al.* 2001; Browning *et al.* 2001; Chaudhry *et al.* 2001; Miller *et al.* 2010; Shi *et al.* 2013)] and two of which are novel (*Colgalt1^fosse/fosse^* and *Tgds^bub/bub^*). After many years of productive forward genetic screens in mice, this work illustrates that there are still new discoveries to be made using this strategy. In addition to generating new models of human disease, the generation of nonlethal hypomorphic alleles can allow the study of gene function *in vivo* in cases where a null allele would prove these analyses to be impossible. We have further refined our inbred screening genetic analysis, demonstrating that analysis of three samples from a mutant phenotype will identify candidate regions with high confidence. Importantly, individually barcoding the samples increases the likelihood that one will find the causal loci in genetically complicated scenarios, such as we experienced with *Colgalt1*^fosse^, *Plod3*^ugli^, and *Fbn2*^timon^. We suggest that our strategy is useful for identifying both recessive and dominant genetic modifiers, and deeper sequencing of affected individuals could reduce the number of candidates to an even more manageable number. This report documents continued improvement of methods that we have developed to accelerate genetic analysis of a screen (Gallego-Llamas *et al.* 2015). These efforts will ultimately act to reduce cost and enable an expeditious transition from genomic to molecular characterization.

## Supplementary Material

Supplemental material is available online at www.g3journal.org/lookup/suppl/doi:10.1534/g3.117.300292/-/DC1.
