# Supplementary material for: Optimizing Genomic Methods for Mapping and Identification of Candidate Variants in ENU Mutagenesis Screens Using Inbred Mice
Source: G3 (Bethesda). 2017 Dec 5;8(2):401–9. doi: 10.1534/g3.117.300292 (PMC5919724; doi:10.1534/g3.117.300292)
Supplement: Supplementary file 1 [file 401FileS1.pdf]

Supplemental file 1: homozygosity mapping

```
#!/usr/bin/env python
import subprocess
import os
import glob
import shutil
import homozygosity_mapping_ub26


##parameters
delim = '\t'
thread_number = '6'

##working dir --- add location of fastq files and a working directory
# working_dir = '/data/atimms/timon_0317'
working_dir = ''
fastq_dir = ''
os.chdir(working_dir)

##programs and files
convert_2_annovar = '/home/atimms/programs/annovar/convert2annovar.pl'
table_annovar = '/home/atimms/programs/annovar/table_annovar.pl'
fasta = '/data/atimms/references/mm10/mm10.fa'
bwa = '/home/atimms/programs/bwa-0.7.12/bwa'
samtools = '/home/atimms/programs/samtools-1.3/samtools'
bcftools = '/home/atimms/programs/bcftools-1.3/bcftools'
picard = '/home/atimms/programs/picard-tools-1.139/picard.jar'
delly = '/home/atimms/programs/delly_v0.7.6_CentOS5.4_x86_64bit'
##files etc
##fastq dictionary, include name of sample and name of fastq files
fq_dict = {}
post_bwa_bam = '.bwa.bam'
sorted_bam = '.bwa_sorted.bam'
mkdup_bam = '.bwa_mkdup.bam'
bamslist_file = 'bams.list'
##name of samtools vcf and input files for annovar
st_vcf = 'timon_0317.vcf.gz'
st_avinputs = []
delly_exclude_regions =
'/data/atimms/references/annovar/mm10/mouse.mm10.excl.tsv'
bedtools = '/home/atimms/programs/bedtools2-master/bin/bedtools'


##annovar parameters
av_genome = 'mm10'
av_buildver = ['-buildver', av_genome]
av_ref_dir = ['/data/atimms/references/annovar/' + av_genome]
##annotate with in house controls and public databases
av_protocol = ['-protocol',
'refGene,rmsk,genomicSuperDups,snp142,generic,generic,generic,generic,generic,ge
neric,generic,generic,generic,generic',
             '-genericdbfile',
'pleather_sc_st.K416.avinput,daredevil.avinput,bub.avinput,J308.st.avinput,J318.
```

```
st.avinput,J320.st.avinput,J327.st.avinput,J328.st.avinput,J329.st.avinput,mgp.v
5.merged.snps_all.dbSNP142.avinput']
av_operation = ['-operation', 'g,r,r,f,f,f,f,f,f,f,f,f,f,f']
av_options = ['-otherinfo', '-remove','-arg', '-splicing 10 ,,,,,,,,,,,,']
#av_options = ['-otherinfo', '-remove', '-vcfinput']

acceptable_chr = ['chr1', 'chr2', 'chr3', 'chr4', 'chr5', 'chr6', 'chr7',
'chr8',
                       'chr9', 'chr10', 'chr11', 'chr12', 'chr13', 'chr14',
'chr15',
                       'chr16', 'chr17', 'chr18', 'chr19', 'chr20', 'chr21',
'chr22',
                       'chr23', 'chr24', 'chr25', 'chrx', 'chry', 'chrX',
'chrY',
                       '1', '2', '3', '4', '5', '6', '7', '8', '9', '10', '11',
'12',
                       '13', '14', '15', '16', '17', '18', '19', '20', '21',
'22', '23',
                       '24', '25', 'x', 'y', 'X', 'Y']

##filtering and hom mapping parameters
##filtering
col_exon = 6
exon_definition = ['exonic', 'splicing']
col_function = 9
syn_definition = 'synonymous SNV'
zygosity_col = 24
cov_col = 26
cov_definition = 5
qual_col = 25
qual_definition = 30
##het snp mapping
genome_fai = '/data/atimms/references/mm10/mm10.fa.fai'
window_size = [1000000,5000000,2000000]
# window_size = [10000000]
step_size = 1000000
info_col = 36
naf_values = [0.8,0.9,0.95]


##check if 's' is a number and return boolean
def is_number(s):
      try:
            float(s)
            return True
      except ValueError:
            return False

##dictionary used to convert operator to .operator command
def operator_dict(op):
      ops = {"==": operator.eq, "!=": operator.ne,">=": operator.ge,"<=":
operator.le, ">": operator.gt,"<": operator.lt,"in": operator.contains}
      return ops[op]
```

```
def filter_ann(working_dir, test_type, input_file, output_file, col_number, op,
comparison):
      """
      method for filtering files by operator and a value, need to provide:
      working directory,
      test type i,e, 'and' or 'or',
      input filename,
      output filename.
      parameters for tests i.e;
      column number
      operator (must be in dict)
      comparison (what to compare value in col number with)
      """
      os.chdir(working_dir)
      print "filtering variants from the file '%s' in an '%s' fashion" %
(input_file,test_type)
      ##variables
      row_number, variants_kept = 0,0                             #keep track of
number of tests and variants kept
      delim = '\t'
      ##open files for reading and writing
      infile = open(input_file, 'rb')
      outfile = open(output_file, 'wb')
      writer = csv.writer(outfile, delimiter=delim)
      ##check row by row
      for row in csv.reader(infile, delimiter=delim):
            row_number += 1
            if row_number == 1:
                  writer.writerow(row)                                  #writes
header to out file
                  header = row                                          #writes
header to header
            else:
                  tests_correct = 0                                     #keep
track of number of correct tests and reset
                  for i in range(len(comparison)):
                        op_func = operator_dict(op[i])
#queries operator dictionary so can perform comparisons
                        col = col_number[i] -1                            #adjust
column number so starts at 1 not 0

                        if row_number == 2:                               #print
info on test completed
                              print "test %r: '%s %s' in column '%s'" % (i,
op[i], comparison[i], header[col])

                        if is_number(comparison[i]):                      #if
comparison is a number
                              # print row[col]
                              if row[col] == 'NA' or row[col] == '.' or row[col]
== '':       #if using na as 'empty' if can't compare number to text so say test
is true
                                    tests_correct += 1
                              elif row[col] == '.':                         # in
gatk sometime coverage = 0 but printed as .
```

```
                                    pass
                              elif op_func(float(row[col]), comparison[i]):
#proper comparison
                                    tests_correct += 1
                        else:
                              if op_func(row[col], comparison[i]):          #if
comparison is not a number
                                    tests_correct += 1
                  ##looks if a 'and' a 'or' or a 'once' test type, and writes
into new file
                  if test_type == "or":                                     #if any
test correct per row
                        if tests_correct > 0:
                              writer.writerow(row)
                              variants_kept += 1
                  elif test_type == "and":                                  #if all
tests correct per row
                        if tests_correct == len(comparison):
                              writer.writerow(row)
                              variants_kept += 1
                  elif test_type == "once":                                 #if one
test correct per row
                        if tests_correct == 1:
                              writer.writerow(row)
                              variants_kept += 1
                  else:
                        print "incorrect test type!!"
      ##print overall numbers
      print "%s variants kept out of %s checked" % (variants_kept, row_number -
1)
      print "filtered variants in file:", output_file
      print "" #empty line
      infile.close()
      outfile.close()



##align with bwa
def align_with_bwa(sample_dict):
      for sample in sample_dict:
            r1_fq = sample_dict[sample][0]
            r2_fq = sample_dict[sample][1]
            print sample, r1_fq, r2_fq
            rg = '@RG\\tID:' + sample + '_RG\\tSM:' + sample + "\\tPL:ILLUMINA"
            pe_bam = sample + post_bwa_bam
            sort_bam = sample + sorted_bam
            pic_dup_bam = sample + mkdup_bam
            bwa_pe = subprocess.Popen([bwa, 'mem', '-M', '-t', '20', '-R', rg,
fasta, r1_fq, r2_fq], stdout=subprocess.PIPE)
            st_sam_bam_pe = subprocess.Popen([samtools, 'view', '-q', '20', '-
b', '-@', '5', '-o', pe_bam, '-'], stdin=bwa_pe.stdout)
            st_sam_bam_pe.wait()
            st_sort_pe = subprocess.Popen([samtools, 'sort','-O', 'bam', '-o',
sort_bam, '-T', sample,'-@', '10', '-m', '10G', pe_bam])
            st_sort_pe.wait()
```

```
            ##mark duplicates
            picard_md = subprocess.Popen(['java', '-Xmx80g', '-jar', picard,
'MarkDuplicates', 'VALIDATION_STRINGENCY=SILENT', 'CREATE_INDEX=true', 'INPUT='
+ sort_bam, 'OUTPUT=' + pic_dup_bam, 'METRICS_FILE=' + sample + '.metrics',
'TMP_DIR=' + working_dir])
            picard_md.wait()

##make list of all bam files to be analyzed
def make_list_of_bams(sample_dict, bam_suffix, bamlist_file):
      with open(bamlist_file, "w") as outfh:
            for sample in sample_dict:
                  bam = sample + bam_suffix
                  outfh.write(bam + '\n')


##call samtools on bamfiles
def variant_calling_samtools(bamlist, final_vcf):
      vcf_temp1 = 'temp_st.vcf.gz'
      vcf_temp2 = 'temp_fb2.vcf.gz'
      stmp = subprocess.Popen([samtools,'mpileup', '-ug','-t', 'DP,DV,DPR', '-
q', '20', '-C', '50', '-f', fasta, '-b', bamlist], stdout=subprocess.PIPE)
      bcft = subprocess.Popen([bcftools,'call', '-vmO', 'z', '-V', 'indels', '-
o', vcf_temp1], stdin=stmp.stdout)
      bcft.wait()
      bcf_index = subprocess.Popen([bcftools, 'index', vcf_temp1])
      bcf_index.wait()
      #split multi-allelic variants calls in separate lines
      bcf_norm1 = subprocess.Popen([bcftools, 'norm', '-m-both', '-o',
vcf_temp2, vcf_temp1])
      bcf_norm1.wait()
      bcf_norm2 = subprocess.Popen([bcftools, 'norm', '-f', fasta, '-O', 'z', '-
o', final_vcf, vcf_temp2])
      bcf_norm2.wait()
      bcf_index = subprocess.Popen([bcftools, 'index', final_vcf])
      bcf_index.wait()

##make avinput files
def convert_to_annovar(vcf):
      con_ann = subprocess.Popen([convert_2_annovar, '-format', 'vcf4', vcf, '-
includeinfo', '-withzyg', '-allsample', '-outfile', 'temp'])
      con_ann.wait()
      temp_files = glob.glob('temp*.avinput')
      for temp_file in temp_files:
            real_file = temp_file[5:]
            os.rename(temp_file, real_file)
            shutil.copy(real_file, str(av_ref_dir[0]))

def run_table_annovar(avinputs):
      for avinput in avinputs:
            av_prefix = avinput.rsplit('.',1)[0]
            command = [table_annovar] + av_buildver + [avinput] + av_ref_dir +
av_protocol + av_operation + av_options + ['-out', av_prefix]
            annovar = subprocess.Popen(command)
            annovar.wait()
```

```
def multianno_to_annotated(avinputs):
	head = ['Chr', 'Start', 'End', 'Ref', 'Alt', 'Func.refGene',
'Gene.refGene', 'GeneDetail.refGene', 'ExonicFunc.refGene', 'AAChange.refGene',
'rmsk', 'genomicSuperDups',
'snp142','pleather','daredevil','bub','J308','J318','J320','J327','J328','J329',
'mgp.v5.snps', 'Zygosity', 'Qual', 'Coverage', 'Chr', 'Pos', 'Filter', 'Ref2',
'Alt2','Qual', 'Filter', 'GT_info', 'Format', 'Info']
	head_out = delim.join(head + ['\n'])
	for avinput in avinputs:
		av_prefix = avinput.rsplit('.',1)[0]
		multianno = av_prefix + '.mm10_multianno.txt'
		annotated = av_prefix + '.annotated.txt'
		with open(multianno, "r") as multi, open(annotated, "w") as final:
			final.write(head_out)
			line_count = 0
			for line in multi:
				line_count += 1
				if line_count > 1:
					final.write(line)


def make_bed_from_ann_txt(infile, outfile):
	with open(outfile, "w") as out_fh, open(infile, "r") as in_fh:
		line_count = 0
		for line in in_fh:
			line_count += 1
			if line_count >1:
				line = line.rstrip().split(delim)
				out_fh.write(delim.join(line[:3] + ['\n']))

##edit bedfile to remove non standard chromosomes
def remove_nonStd_chr(infile, outfile):
	fh = open(infile, 'r')
	outfh = open(outfile, 'w')
	for line in fh:
		line = line.split(delim)
		if line[0] in acceptable_chr:
			outfh.write(delim.join(line))
	fh.close()
	outfh.close()


##divide genome into windows
def make_windows(working_dir, genome_fai, window_size, step_size):
	os.chdir(working_dir)
	genome = genome_fai.split('/')[-1].split('.')[0]
	genome_window_bed = genome + '_' + str(window_size / 1000) + 'kb_' +
str(step_size / 1000) + 'kb' + '.bed'
	print 'bed file %s created from file: %s with window size %r and step size
%r' % (genome_window_bed, genome_fai, window_size, step_size)
	print ''
	##run bedtools makewindows without pybedtools installed
	command = bedtools + ' makewindows -g ' + genome_fai + ' -w ' +
str(window_size) + ' -s ' + str(step_size) + ' > ' + 'bed.temp'
	subprocess.call(command, shell=True)
```

```
	##filters chromosomes if required
	remove_nonStd_chr('bed.temp', genome_window_bed)
	##if you don't require any filtering
	#shutil.copy('bed.temp', genome_window_bed)
	return genome_window_bed

##make bed file from ann.txt contains: chr,start,end,zygosity,novel_allele_freq
def make_bed_from_ann(working_dir, var_caller, filename, zygosity_col, 
last_col):
	os.chdir(working_dir)
	fh = open(filename, 'r')
	outfile = filename.split(".", 1)[0] + '.bed'
	outfh = open(outfile, 'w')
	line_count = 0
	kept_count = 0
	for line in fh:
		line_count += 1
		if line_count > 1:
			line = line.split(delim)
			info = line[last_col - 1].split(':')
			#print info
			if var_caller == 'gatk' or var_caller == 'gatk_hc':
				if len(info[1].split(',')) != 2: #gives snps with mutiple 
alleles naf of 0
					non_ref_allele, total_alleles = 0,0
				elif info[2] == '.':
					non_ref_allele, total_alleles = 0,0
				else:
					non_ref_allele = float(info[1].split(',')[1])
					total_alleles = int(info[2])


			elif var_caller == 'samtools':
				if len(info[1].split(',')) != 3: #gives snps with mutiple 
alleles naf of 0
					non_ref_allele, total_alleles = 0,0
				else:
					non_ref_allele = float(info[3])
					total_alleles = int(info[2])


			elif var_caller == 'freebayes':
				if ',' in info[4]: #gives snps with mutiple alleles naf 
of 0
					non_ref_allele, total_alleles = 0,0
				else:
					non_ref_allele = float(info[4])
					total_alleles = int(info[1])

			else:
				print "doofus: %s isn't a variant caller" % var_caller
			if total_alleles == 0:
				naf = 0
			else:
				naf = non_ref_allele / total_alleles
```

```
                #print var_caller, info, non_ref_allele, total_alleles, naf

                if naf > 0.1:           #prevents very low frequency SNPs and
those we've filtered
                        zygosity = line [zygosity_col -1]
                        line_out = [line [0], str(int(line [1]) - 1), line [2],
zygosity, str(naf), '\n']
                        outfh.write(delim.join(line_out))
                        kept_count += 1
                        #print 'kept'
                #else:
                        #print info, naf
        print 'bed file %s created from %s' % (outfile, filename)
        print '%r variants checked and %r kept' % (line_count -1, kept_count)
        print ''
        fh.close()
        outfh.close()

##take bedfile with zygosity in 4th col and write a het and hom temp file
def split_het_hom(working_dir, filename):
        fh = open(filename, 'r')
        het_fh = open('het.temp', 'w')
        hom_fh = open('hom.temp', 'w')
        line_count = 0
        het_count = 0
        hom_count = 0
        for line in fh:
                line_count += 1
                line = line.split(delim)
                if line[3] == 'het':
                        het_fh.write(delim.join(line))
                        het_count += 1
                elif line[3] == 'hom':
                        hom_fh.write(delim.join(line))
                        hom_count += 1
                else:
                        print 'issue with snp on line', line_count
        fh.close()
        het_fh.close()
        hom_fh.close()
        print 'for file %s we have %r snps, of which %r are het and %r are hom' %
(filename, line_count, het_count, hom_count)
        print ''

##combines multiple beds
##'chr:start-end' must match and be sorted in same way
##will only add last col, so need to adjust if need more
def combine_multiple_bed(bed_list, outfile):
        results = []
        no_of_bed = 0
        ##loop through bedfiles and add to lists
        for bed in bed_list:
                fh = open(bed, 'r')
                no_of_bed += 1
```

```
            line_number = -1
            for line in fh:
                  line_number += 1
                  line = line.strip('\n').split(delim)
                  ##if first file add all columns
                  if no_of_bed == 1:
                        results.append(line)
                  ##if not first file check 'chr:start-end' and add last column
                  if no_of_bed >1:
                        if results[line_number][:3] == line[:3]:
                              results[line_number].append(line[-1])
            fh.close()
      ##write to outfile
      outfh = open(outfile, 'w')
      for i in range(len(results)):
            outfh.write(delim.join(results[i]) + '\n')
      outfh.close()
      print "combined the %r bedfiles %s" % (no_of_bed, ','.join(bed_list))
      print "checked %r and printed %r lines" % (line_number + 1 , len(results))

##make het and hom count bedgraphs for specified window size
def het_and_hom_bed(working_dir, genome_and_window, filename):
      os.chdir(working_dir)
      print 'making het and hom bedfiles for:', filename
      split_het_hom(working_dir, filename)
      sample = filename.split(".", 1)[0]
      window_bed = genome_and_window + '.bed'
      hom_count_bed = sample + '_' + genome_and_window + '_hom_count.bedgraph'
      het_count_bed = sample + '_' + genome_and_window + '_het_count.bedgraph'
      ##pbt version
      # a = pbt.BedTool(genome_and_window + '.bed')
      # b = pbt.BedTool('hom.temp')
      # c = pbt.BedTool('het.temp')
      # a_with_b = a.intersect(b, c=True).moveto(hom_count_bed)
      # a_with_c = a.intersect(c, c=True).moveto(het_count_bed)
      ##bedtools version
      #bedtools intersect -a A.bed -b B.bed -c
      with open(hom_count_bed, "w") as hom_fh:
            hom_bt_intersect = subprocess.Popen([bedtools, 'intersect', '-a',
window_bed, '-b', 'hom.temp', '-c'], stdout=hom_fh)
            hom_bt_intersect.wait()
      with open(het_count_bed, "w") as het_fh:
            hom_bt_intersect = subprocess.Popen([bedtools, 'intersect', '-a',
window_bed, '-b', 'het.temp', '-c'], stdout=het_fh)
            hom_bt_intersect.wait()
      return hom_count_bed, het_count_bed

##count hom and het, and hom percentage in specified window
def count_and_percentage(working_dir, genome_and_window, filename):
      os.chdir(working_dir)
      print 'calculating hom count, het count and hom percentage for:', filename
      hom_count_bed, het_count_bed = het_and_hom_bed(working_dir,
genome_and_window, filename)
      combine_multiple_bed([hom_count_bed, het_count_bed], 'hom_het.temp')
      fh = open('hom_het.temp', 'r')
```

```
    sample = filename.split(".", 1)[0]
    outfile = sample + '_' + genome_and_window + '_hom_percentage.bedgraph'
    outfh = open(outfile, 'w')
    for line in fh:
        line = line.strip('\n').split(delim)
        if line[3] == '0':
            hom_percentage = 0
        else:
            hom_percentage = float(line[3]) / (float(line[3]) +
float(line[4]))
        outfh.write(delim.join(line[:3]) + delim + str(hom_percentage) +
'\n')
    fh.close()
    outfh.close()
    print 'generated files:'
    print 'hom_count:', hom_count_bed
    print 'het_count:', het_count_bed
    print 'hom_percentage:', outfile


##novel allele frequency within windows
def naf_in_window(working_dir, genome_and_window, bed_file):
    os.chdir(working_dir)
    print 'calculating novel allele freq within windows for file:', bed_file
    sample = bed_file.split(".", 1)[0]
    ##bedtools interect window bed and sample bed
    # a = pbt.BedTool(genome_and_window + '.bed')
    # b = pbt.BedTool(bed_file)
    # a_with_b = a.intersect(b, wa=True, wb=True).moveto(sample + '_naf.temp')
    ##bedtools version
    window_bed = genome_and_window + '.bed'
    #bedtools intersect -a A.bed -b B.bed -c
    with open(sample + '_naf.temp', "w") as naf_fh:
        hom_bt_intersect = subprocess.Popen([bedtools, 'intersect', '-a',
window_bed, '-b', bed_file, '-wa', '-wb'], stdout=naf_fh)
        hom_bt_intersect.wait()
    ##make dictionary: key = chr:start-end and value a list of naf values
    naf_dict = {}
    fh = open(sample + '_naf.temp', 'r')
    for line in fh:
        line = line.strip('\n').split(delim)
        chr_start_end = delim.join(line[:3])
        naf_value = float(line[-1])
        if naf_dict.has_key(chr_start_end):
            naf_dict[chr_start_end].append(naf_value)
        else:
            naf_dict[chr_start_end] = []
            naf_dict[chr_start_end].append(naf_value)
    fh.close()

    ##make list of lists containing chr,start,end,average_naf, snp#
    list_of_lists = []
    for cse in naf_dict.keys():
        split_cse = cse.split(delim)
        average_naf = [sum(naf_dict[cse])/float(len(naf_dict[cse]))]
```

```
            total_snps = [len(naf_dict[cse])]
            outlist = split_cse + average_naf + total_snps
            outlist[1] = int(outlist[1])
            outlist[2] = int(outlist[2])
            list_of_lists.append(outlist)
      list_of_lists.sort(key = operator.itemgetter(0, 1))

      ##print bed file with average_naf
      outfh = open(sample + '_' + genome_and_window + '_naf.bedgraph', 'w')
      for line in list_of_lists:
            for i in range(len(line)):
                  line[i] = str(line[i])
            outfh.write(delim.join(line) + '\n')
      outfh.close()

##total snp number within windows (for testing)
def total_snp_in_window(working_dir, genome_and_window, bed_file):
      os.chdir(working_dir)
      print 'caluclating total snp number within windows for file:', bed_file
      sample = bed_file.split(".", 1)[0]
      ##bedtools interect window bed and sample bed
      # a = pbt.BedTool(genome_and_window + '.bed')
      # b = pbt.BedTool(bed_file)
      # a_with_b = a.intersect(b, wa=True, wb=True).moveto(sample + '_naf.temp')
      ##bedtools version
      window_bed = genome_and_window + '.bed'
      #bedtools intersect -a A.bed -b B.bed -c
      with open(sample + '_naf.temp', "w") as naf_fh:
            hom_bt_intersect = subprocess.Popen([bedtools, 'intersect', '-a',
window_bed, '-b', bed_file, '-wa', '-wb'], stdout=naf_fh)
            hom_bt_intersect.wait()

      ##make dictionary: key = chr:start-end and value a list of naf values
      naf_dict = {}
      fh = open(sample + '_naf.temp', 'r')
      for line in fh:
            line = line.strip('\n').split(delim)
            chr_start_end = delim.join(line[:3])
            naf_value = float(line[-1])
            if naf_dict.has_key(chr_start_end):
                  naf_dict[chr_start_end].append(naf_value)
            else:
                  naf_dict[chr_start_end] = []
                  naf_dict[chr_start_end].append(naf_value)
      fh.close()

      ##make list of lists containing chr,start,end,total_snp, snp#
      list_of_lists = []
      for cse in naf_dict.keys():
            split_cse = cse.split(delim)
            total_snps = [len(naf_dict[cse])]
            outlist = split_cse + total_snps
            outlist[1] = int(outlist[1])
            outlist[2] = int(outlist[2])
            list_of_lists.append(outlist)
```

```
      list_of_lists.sort(key = operator.itemgetter(0, 1))

      ##print bed file with total snp number
      outfh = open(sample + '_' + genome_and_window + '_snp_number.bedgraph',
'w')
      for line in list_of_lists:
            for i in range(len(line)):
                  line[i] = str(line[i])
            outfh.write(delim.join(line) + '\n')
      outfh.close()


##method to combine hom mapping data i.e. bedgraphs
##suply working dir, file prefix name, and genome and window information
def combine_bedgraphs_for_r(working_directory, prefix, genome_window):
      chr_wanted = ['chr1', 'chr2', 'chr3', 'chr4', 'chr5', 'chr6', 'chr7',
'chr8', 'chr9', 'chr10',
            'chr11', 'chr12', 'chr13', 'chr14', 'chr15', 'chr16', 'chr17',
'chr18', 'chr19', 'chr20',
            'chr21', 'chr22', 'chr23', 'chr24', 'chr25','1', '2', '3', '4', '5',
'6', '7', '8', '9',
            '10', '11', '12', '13', '14', '15', '16', '17', '18', '19', '20',
'21', '22', '23', '24', '25']
      snp_count = prefix + '_' + genome_window + '_snp_number.bedgraph'
      het_count = prefix + '_' + genome_window + '_het_count.bedgraph'
      hom_count = prefix + '_' + genome_window + '_hom_count.bedgraph'
      hom_perc = prefix + '_' + genome_window + '_hom_percentage.bedgraph'
      naf_ph = prefix + '_' + genome_window + '_naf.bedgraph'
      combined_r_file = prefix + '_' + genome_window +
'_combined_hom_mapping.txt'
      with open(combined_r_file, "w") as r_file:
            r_file.write(delim.join(['chr', 'start', 'end', 'value',
'chromosome', 'analysis', '\n']))
            with open(hom_count, "r") as homc:
                  for line in homc:
                        line = line.strip('\n').split(delim)
                        line = [line[0].replace('chr','')] + line[1:] #removes
chr from start of line
                        ##remove chromosomes we're not interested in
                        if line[0] in chr_wanted:
                              start = int(line[1])
                              end = int(line[2])
                              midpoint = start + ((end - start) / 2)
                              r_file.write(delim.join(line[:4] + [str(midpoint),
'hom count', '\n']))
            with open(hom_perc, "r") as homp:
                  for line in homp:
                        line = line.strip('\n').split(delim)
                        line = [line[0].replace('chr','')] + line[1:] #removes
chr from start of line
                        if line[0] in chr_wanted:
                              start = int(line[1])
                              end = int(line[2])
                              midpoint = start + ((end - start) / 2)
```

```
                        r_file.write(delim.join(line[:4] + [str(midpoint),
'hom percentage', '\n']))
            with open(naf_ph, "r") as naf:
                  for line in naf:
                        line = line.strip('\n').split(delim)
                        line = [line[0].replace('chr','')] + line[1:] #removes
chr from start of line
                        if line[0] in chr_wanted:
                              start = int(line[1])
                              end = int(line[2])
                              midpoint = start + ((end - start) / 2)
                              r_file.write(delim.join(line[:4] + [str(midpoint),
'average naf', '\n']))
##call methods, samtools, annovar and format multianno


##map with bwa and process with samtools etc
align_with_bwa(fq_dict)
make_list_of_bams(fq_dict, mkdup_bam, bamslist_file)
variant_calling_samtools(bamslist_file, st_vcf)
convert_to_annovar(st_vcf)
run_table_annovar(st_avinputs)
multianno_to_annotated(st_avinputs)

##filter vars and hom mapping

#filter variants for candidates snps
for sample in fq_dict:
      ##exonic_variants
      filter_ann(working_dir, "or", sample + '.annotated.txt' , sample +
"_1.temp", [col_exon, col_exon], ['==','=='],
[exon_definition[0],exon_definition[1]])
      ##remove synonymous
      filter_ann(working_dir, "and", sample + "_1.temp", sample + "_2.temp",
[col_function], ['!='], [syn_definition])
      ##remove if in dbsnp, sanger, or other mouse line
      filter_ann(working_dir, "and", sample + "_2.temp", sample + "_3.temp",
[13,14,15,16,17,18,19,20,21,22,23],
['==','==','==','==','==','==','==','==','==','==','=='],
['','','','','','','','','','',''])
      ##keep if hom
      filter_ann(working_dir, "and", sample + "_3.temp", sample +
'.hom_exonic_rare.xls', [zygosity_col], ['=='], ['hom'])
      ##filter variants by coverage and quality
      filter_ann(working_dir, "and", sample + '.hom_exonic_rare.xls', sample +
'.hom_exonic_rare_qual_filtered.xls', [cov_col,qual_col], ['>=','>='],
[cov_definition,qual_definition])


##homozygosity mapping
for ws in window_size:
      for sample in fq_dict:
            ##remove if in dbsnp, sanger, other ped or rmsk
            filter_ann(working_dir, "and", sample + '.annotated.txt', sample +
"11.temp", [11,12,13,14,15,16,17,18,19,20,21,22,23],
```

```
['==','==','==','==','==','==','==','==','==','==','==','==','=='],
['','','','','','','','','','','','',''])
            # filtering_annotated.filter(working_dir, "and", sample +
'.annotated.txt', sample + "11.temp", [11,12,23], ['==','==','=='], ['','',''])

            ##filter variants by coverage and quality
            filter_ann(working_dir, "and", sample + "11.temp", sample +
'.hom_temp.txt', [cov_col,qual_col], ['>=','>='],
[cov_definition,qual_definition])

            #make bed file with windows and returns genome name and window size
variable
            genome_and_window = make_windows(working_dir, genome_fai, ws,
step_size).split('.')[0]

            ##make bed file from variants
            make_bed_from_ann(working_dir, 'samtools', sample +
'.hom_temp.txt', zygosity_col, info_col)
            ##hom and het count and hom percentage
            count_and_percentage(working_dir, genome_and_window, sample +
'.bed')
            ##naf
            naf_in_window(working_dir, genome_and_window, sample + '.bed')
            ##total snp number
            total_snp_in_window(working_dir, genome_and_window, sample + '.bed')

            ##combine bedgraphs for graphing in r
            combine_bedgraphs_for_r(working_dir, sample, genome_and_window)
```

Supplemental Table 1. Recombination map of the ENU-induced *fosse* locus on chromosome 8.

Embryo ID	Sex	*Ddx60*	*Colgalt1*	*Jak3*	*Ces1e*	*Hsf4*	Embryo Phenotype
K407.0003.4	F	MUT/MUT	MUT/MUT	MUT/MUT	WT/WT	WT/WT	*fosse*
K407.0009.7	M	MUT/MUT	MUT/MUT	N/A	WT/WT	WT/WT	*fosse*
K421.0003.1	M	MUT/MUT	MUT/MUT	MUT/MUT	WT/MUT	WT/MUT	*fosse*
K421.0003.5	M	WT/MUT	MUT/MUT	MUT/MUT	MUT/MUT	MUT/MUT	*fosse*
K421.0018.8	F	MUT/MUT	MUT/MUT	MUT/MUT	MUT/MUT	WT/MUT	*fosse*
K421.0035.7	F	WT/MUT	MUT/MUT	MUT/MUT	MUT/MUT	MUT/MUT	*fosse*
K422.0002.5	M	MUT/MUT	MUT/MUT	MUT/MUT	WT/WT	WT/WT	*fosse*
K422.0002.6	F	MUT/MUT	MUT/MUT	MUT/MUT	WT/WT	WT/WT	*fosse*
**K421.0009.4**	F	MUT/MUT	MUT/MUT	MUT/MUT	MUT/MUT	MUT/MUT	*fosse* with cleft palate
**K421.0018.7**	M	MUT/MUT	MUT/MUT	MUT/MUT	MUT/MUT	MUT/MUT	*fosse* with cleft palate
**K421.0019.5**	M	WT/MUT	MUT/MUT	MUT/MUT	MUT/MUT	MUT/MUT	*fosse* with cleft palate
**K421.0022.3**	F	MUT/MUT	MUT/MUT	MUT/MUT	MUT/MUT	MUT/MUT	*fosse* with cleft palate
**K421.0022.4**	M	MUT/MUT	MUT/MUT	MUT/MUT	MUT/MUT	MUT/MUT	*fosse* with cleft palate
K421.0035.8	F	WT/MUT	MUT/MUT	MUT/MUT	MUT/MUT	MUT/MUT	*fosse* with cleft palate
K433.0006.4	M	WT/WT	MUT/MUT	MUT/MUT	WT/WT	WT/WT	*fosse* with cleft palate
K433.0014.2	F	WT/WT	MUT/MUT	MUT/MUT	WT/WT	WT/WT	*fosse* with cleft palate
K421.0009.5	F	MUT/MUT	MUT/MUT	MUT/MUT	MUT/MUT	MUT/MUT	*fosse* with exencephaly
K421.0019.4	M	MUT/MUT	MUT/MUT	MUT/MUT	N/A	WT/MUT	*fosse* with exencephaly
K421.0028.3	M	MUT/MUT	MUT/MUT	MUT/MUT	MUT/MUT	MUT/MUT	*fosse* with exencephaly
**K421.0060.3**	F	MUT/MUT	MUT/MUT	MUT/MUT	WT/MUT	WT/MUT	*fosse* with exencephaly
K433.0006.5	M	WT/WT	MUT/MUT	MUT/MUT	WT/WT	WT/WT	*fosse* with exencephaly
K407.0032.4	M	WT/WT	WT/WT	WT/WT	WT/WT	WT/WT	*ugli*
**K407.0034.2**	M	WT/MUT	WT/MUT	WT/MUT	WT/WT	WT/WT	*ugli*
**K407.0034.7**	F	WT/WT	WT/MUT	WT/WT	WT/WT	WT/WT	*ugli*

All sample ID #s in bold type were included in WGS analysis

Supplemental Table 2. Genotyping data for *ugli* candidate variants.

Embryo ID	Sex	*Pnpla3* Chr 15	*Gm15800* Chr 5	*Plod3* Chr 5	Phenotype
K407.0032.4	M	?	MUT/MUT	MUT/MUT	*ugli* exencephaly
**K407.0034.2**	M	MUT/MUT	WT/MUT	MUT/MUT	*ugli* exencephaly
**K407.0034.7**	F	WT/MUT?	MUT/MUT	MUT/MUT	*ugli* exencephaly
K407.0003.4	F	WT/MUT?	MUT/MUT	WT/MUT	*fosse*
K407.0009.7	M	WT/WT	WT/WT	WT/WT	*fosse*
K421.0009.5	F	WT/WT	WT/WT	WT/WT	*fosse* exencephaly
K421.0019.4	M	WT/WT	WT/WT	WT/WT	*fosse* exencephaly
K421.0028.3	M	WT/WT	WT/MUT	WT/WT	*fosse* exencephaly
**K421.0060.3**	F	WT/WT	WT/WT	WT/WT	*fosse* exencephaly

All sample ID #s in bold type were included in WGS analysis

Supplemental Table 3. Genotyping of *daredevil* and *bub* candidate variants from affected and unaffected littermates.

daredevil candidate genotyping				
**Embryo ID**	***Pgm5***	***Kif20b***	**--------------**	**Embryo Phenotype**
**K416_0006_5**	MUT/MUT	MUT/MUT	--------------	*daredevil*
**K416_0007_9**	MUT/MUT	MUT/MUT	--------------	*daredevil*
K416_0036_1	MUT/MUT	MUT/MUT	--------------	*daredevil*
K417_0013_5	MUT/MUT	MUT/MUT	--------------	*daredevil*
**K417_0024_8**	WT/MUT	MUT/MUT	--------------	*daredevil*
K417_0024_9	WT/MUT	MUT/MUT	--------------	*daredevil*
K417_0024_10	WT/MUT	MUT/MUT	--------------	*daredevil*
K417_0032_3	MUT/MUT	MUT/MUT	--------------	*daredevil*
K417_0044_1	MUT/MUT	MUT/MUT	--------------	*daredevil*
K417_0044_2	MUT/MUT	MUT/MUT	--------------	*daredevil*
K417_0051_1	WT/MUT	MUT/MUT	--------------	*daredevil*
K417_0062_1	MUT/MUT	MUT/MUT	--------------	*daredevil*
K416_0006_4	WT/WT	WT/WT	--------------	Unaffected
K416_0007_4	WT/MUT	WT/MUT	--------------	Unaffected
K417_0032_4	WT/WT	WT/MUT	--------------	Unaffected
K417_0044_4	WT/MUT	WT/MUT	--------------	Unaffected
bub **candidate genotyping**				
**Embryo ID**	***Dact1***	***Myh6***	***Tgds***	**Embryo Phenotype**
K402_0014_1	MUT/MUT	MUT/MUT	MUT/MUT	*bub*
K402_0014_5	WT/MUT	WT/WT	MUT/MUT	*bub*
**K402_0014_6**	WT/MUT	MUT/MUT	MUT/MUT	*bub*
K402_0015_3	WT/WT	MUT/MUT	MUT/MUT	*bub*
K402_0015_4	MUT/MUT	WT/WT	MUT/MUT	*bub*
**K402_0035_1**	WT/MUT	MUT/MUT	MUT/MUT	*bub*
**K402_0035_2**	MUT/MUT	MUT/MUT	MUT/MUT	*bub*
K402_0040_10	WT/WT	WT/WT	MUT/MUT	*bub*
K402_0014_3	WT/WT	WT/MUT	WT/WT	Unaffected
K402_0015_2	WT/MUT	WT/MUT	WT/MUT	Unaffected
K402_0035_5	WT/MUT	MUT/MUT	WT/MUT	Unaffected
K402_0035_6	WT/WT	WT/WT	WT/WT	Unaffected

All sample ID #s in bold type were included in WGS analysis

Supplemental Table 4. Genotyping of *timon* mutants across two critical intervals on chromosomes 7 and 18.

**Chr7**	1	2	3	4	5	6	7	8	9	10	11	12	13	14	15	16	17	18
**Gene ID**																		
Ccp110	HET	WT	HET	HET	HET	HET	WT	HET	MUT	HET	MUT	MUT	MUT					
Sult1a1	HET	WT	HET	HET	HET	HET	HET	HET	MUT	HET	MUT	MUT	MUT					
**Chr18**																		
**Gene ID**																		
Chst9/Chd2	WT	WT	MUT	MUT	MUT	WT	HET	HET	MUT	MUT	HET	HET	MUT					
Pcdhb10	WT	WT	MUT	MUT	MUT	WT	HET	HET	MUT	MUT	HET	MUT	MUT					
1700011I03Rik	WT	WT	MUT	MUT	MUT	MUT	MUT	MUT	MUT	MUT	MUT	MUT	MUT	MUT	MUT	MUT	MUT	MUT
***Fbn2* Deletion**	WT	WT	MUT	MUT	MUT	MUT	MUT	MUT	MUT	MUT	MUT	MUT	MUT	MUT	MUT	MUT	MUT	MUT
Pdgfrb	WT	WT	MUT	MUT	MUT	MUT	MUT	MUT	MUT	MUT	MUT	MUT	MUT	MUT	MUT	MUT	MUT	MUT
miR466p	WT	WT	MUT	MUT	MUT	MUT	MUT	MUT	MUT	MUT	MUT	MUT	MUT	MUT	MUT	MUT	MUT	MUT
						SEQ			SEQ				SEQ					

Chromosome 7 critical interval = 32.9 Mb

Chromosome 18 critical interval = 52 Mb

Deletion on chromosome 18 = 58,012,626-58,014,322, < 400 kb from the *1700011I03Rik* variant, 3 Mb from *Pdgfrb* variant

SEQ: Genomic DNA was submitted for WGS
